# Functionality, satisfaction, and global impression of change with ubrogepant for the acute treatment of migraine in triptan insufficient responders: a post hoc analysis of the ACHIEVE I and ACHIEVE II randomized trials

**DOI:** 10.1186/s10194-022-01419-7

**Published:** 2022-04-25

**Authors:** Richard B. Lipton, Rashmi B. Halker Singh, Dennis A. Revicki, Sihui Zhao, Anand R. Shewale, Jordan E. Lateiner, David W. Dodick

**Affiliations:** 1 Albert Einstein College of Medicine, Bronx, NY USA; 2 Mayo Clinic, Phoenix, AZ USA; 3 Outcomes Research Consulting, Sarasota, FL USA; 4 AbbVie, Madison, NJ USA; 5 AbbVie, 2525 Dupont Dr., Irvine, CA 92612 USA

**Keywords:** ACHIEVE, Calcitonin gene–related peptide receptor antagonist, Migraine, Patient-reported outcomes, Functionality, Satisfaction, Triptan

## Abstract

**Background:**

Triptans are the first-line option for the acute treatment of migraine attacks; however, triptans are contraindicated in people with certain underlying cardiovascular risk factors and are associated with inadequate efficacy or poor tolerability in some individuals. Ubrogepant is an oral calcitonin gene–related peptide receptor antagonist approved for the acute treatment of migraine.

**Methods:**

This post hoc analysis of the phase 3 ACHIEVE trials examined the impact of ubrogepant on the Functional Disability Scale (FDS), satisfaction with medication, and Patient Global Impression of Change (PGIC) in participants who were self-reported triptan insufficient responders (TIRs), defined as those who are unable to take triptans due to contraindications, tolerability issues, or insufficient efficacy. Responder definitions for the FDS, satisfaction measures, and PGIC were based on qualitative interpretation of the respective response options for the pooled ubrogepant 50 mg and placebo groups.

**Results:**

In the pooled analysis population (*n* = 1799), 451 (25%) participants were TIRs, with most (80%) reporting insufficient efficacy with triptan use. A significantly higher proportion of TIRs treated with ubrogepant vs placebo reported being able to function normally from 2 to 8 h post dose (*P* < 0.05). Notably, significance was demonstrated at the time of the primary outcome assessments (2 h post dose), where rates of normal function were 38% for ubrogepant vs 29% for placebo (*P* = 0.048). A greater proportion of TIRs in the ubrogepant arm vs the placebo arm were satisfied with treatment at 2 (33% vs 21%, *P* = 0.006) and 24 h (58% vs 28%, *P* < 0.001) and indicated that their migraine improved at 2 h vs placebo (30% vs 18%, *P* = 0.006). Results were generally similar in the insufficient efficacy subpopulation of TIRs as in the overall TIRs group. Ubrogepant was safe and well tolerated in TIRs, with no new safety signals identified.

**Conclusions:**

In people with migraine who are TIRs, individuals treated with ubrogepant had favorable 2-h outcomes, as measured by the FDS, satisfaction with medication, and PGIC, compared with placebo.

**Trial registration:**

ClinicalTrials.gov: NCT02828020 (ACHIEVE I), registered July 11, 2016; NCT02867709 (ACHIEVE II), registered August 16, 2016.

## Introduction

Migraine is a complex, chronic neurological disease that is a leading cause of years lived with disability globally [1, 2]. Symptoms associated with migraine can be severe and often debilitating [3, 4]. Repeated attacks can ultimately have a negative impact on relationships, finances, and overall well-being [4, 5]. The burden associated with migraine remains high and severely impacts an individual’s health-related quality of life (HRQoL) [4, 5].

Triptans have established efficacy and are widely prescribed as first-line acute treatment for moderate or severe migraine attacks [6, 7]. Triptans constrict arteries and have been associated with ischemic events [8, 9]; they are contraindicated in persons with symptomatic cardiovascular (CV) disease (e.g., history of myocardial infarction or stroke, peripheral vascular disease, uncontrolled hypertension) and carry a warning or precaution for use in those with CV risk factors [10, 11]. A claims-based analysis found that at least 20% of individuals with migraine had at least 1 significant CV contraindication to triptans and an additional 25% of individuals had 2 or more CV risk factors consistent with warnings and precautions to triptan use [12].

Although triptans are an effective acute treatment option, many people report dissatisfaction with triptans due to insufficient efficacy or issues with tolerability. Results from the Migraine in America Symptoms and Treatment study found that 44% of individuals who responded to the survey reported inadequate treatment response with triptans [7]. Furthermore, individuals with insufficient response with triptans experience significantly decreased HRQoL and reduced work productivity due to greater monthly headache days and migraine severity, as well as 1.5 to 3 times higher total all-cause and migraine-related healthcare resource utilization costs, respectively [13, 14]. Recently, new acute treatment options have emerged, including medications that target the calcitonin gene–related peptide (CGRP) receptor.

CGRP is a potent vasodilator and an inflammatory mediator that has been implicated as a key element in the pathogenesis of migraine [15]. Ubrogepant is an oral, selective CGRP receptor antagonist (gepant) approved for the acute treatment of migraine with or without aura in adults [16]. The safety and efficacy of ubrogepant have been demonstrated in the ACHIEVE I and II single-attack phase 3 trials, as well as in a long-term (52-week) extension study [17–19]. Gepants can provide an effective alternative acute treatment option for those who are unable to take triptans due to contraindications, tolerability issues, or insufficient efficacy [20–22]. The collective term for these individuals is triptan insufficient responders (TIRs).

Assessing patient-reported outcomes (PROs) is an important aspect of measuring the burden of disease and the benefits of treatment [6, 23, 24]. In an analysis of data from ACHIEVE I and II, a significantly higher proportion of participants randomized to ubrogepant were satisfied with study medication and reported being able to function normally 2 h post dose compared with placebo [25]. However, these outcomes were evaluated in the entire trial sample and not in individuals who were TIRs, a group most likely to receive ubrogepant [26]. The purpose of this post hoc analysis of pooled data from ACHIEVE I and II was to examine the impact of ubrogepant on functional disability, satisfaction with medication, and global impression of change in the subgroup of participants who were classified as TIRs.

## Methods

### Trial design

The ACHIEVE I (NCT02828020) and ACHIEVE II (NCT02867709) trials were phase 3, randomized, double-blind, multicenter, single-attack trials that evaluated the clinical efficacy and safety of ubrogepant vs placebo for the acute treatment of migraine [17, 18]. Eligibility criteria and methods of the ACHIEVE trials have been reported previously in detail [17, 18]. Briefly, adults with a history of migraine with or without aura were randomized 1:1:1 to receive placebo, ubrogepant 50 mg, or ubrogepant 100 mg in ACHIEVE I, and placebo, ubrogepant 25 mg, or ubrogepant 50 mg in ACHIEVE II to treat a single attack.

Participants were asked to take their assigned study medication as soon as possible (no later than 4 h after headache onset) to treat a qualifying migraine attack; a qualified attack was defined as moderate to severe headache pain accompanied by at least 1 migraine-associated symptom (photophobia, phonophobia, or nausea). An optional second dose of study medication or a rescue medication (eg, acetaminophen, nonsteroidal anti-inflammatory drugs, opioid, antiemetic, triptan) was allowed for the treatment of moderate or severe headache pain starting from 2 to 48 h after the initial dose. For those who opted to take a second dose of study medication, participants in the ubrogepant groups were randomized to receive either placebo or ubrogepant (dose same as initial dose) for the optional second dose, whereas all participants in the placebo group received placebo for the optional second dose [17, 18]. The ACHIEVE I and ACHIEVE II trials were conducted in conformance with the Declaration of Helsinki principles, or US laws and regulations, whichever afforded the greater protection to the individual. Trial protocols were approved by each individual research center’s institutional review board. Written informed consent was obtained from all participants prior to initiation of trial procedures. All authors had full access to all data from both studies.

### Outcome measures

The coprimary and secondary efficacy outcomes from ACHIEVE I and II have been previously published [17, 18]. At screening, physicians interviewed participants on their historical response to triptans. Based on these data, participants were categorized into 3 groups: triptan responders, triptan insufficient responders (TIRs), and triptan naive. Triptan responders included those who reported currently using a triptan or had used a triptan in the past 6 months and achieved pain freedom at 2 h post dose on more than half of triptan-treated attacks. TIRs included those currently using a triptan or who used a triptan in the past 6 months and did not achieve pain freedom at 2 h post dose on more than half of the occasions it was taken, or no longer use a triptan because of lack of efficacy and/or side effects, or never used a triptan because of warnings, precautions, or contraindications (eg, CV disease, specifically in individuals with coronary artery disease, a history of stroke, peripheral vascular disease, or chronically uncontrolled hypertension). Triptan-naive participants reported no prior exposure to triptans and did not have contraindications to triptans.

Randomization was stratified by previous experience with triptans and current use of preventive medication for migraine (yes/no). This post hoc analysis reports the results of prespecified efficacy analyses of PRO measures for the pooled ubrogepant 50 mg and placebo groups from each ACHIEVE trial: the Functional Disability Scale (FDS), participant satisfaction with study medication, and Patient Global Impression of Change (PGIC) scale [17, 18]. On the FDS, participants rated their functional ability using a single-item, 4-point response scale ranging from 0 to 3 (0 = no disability, able to function normally; 1 = mildly impaired, can still do everything but with difficulty; 2 = moderately impaired, unable to do some things, 3 = severely impaired, cannot do all or most things, bed rest may be necessary) before dosing and at 1, 2, 4, and 8 h after initial dose. Responders on the FDS were defined as participants who provided a score of 0 (no disability, able to function normally). Satisfaction with study medication was rated on a single-item, 7-point response scale ranging from 0 to 6 (0 = extremely satisfied, 1 = satisfied, 2 = slightly satisfied, 3 = neither satisfied nor dissatisfied, 4 = slightly dissatisfied, 5 = dissatisfied, 6 = extremely dissatisfied) at 2 and 24 h after dosing. Responders were defined as participants who reported scores of 1 (satisfied) or 0 (extremely satisfied) at 2 and 24 h after dosing. The timepoints at which each outcome was collected and analyzed were prespecified, and no data were collected at any other timepoints.

Participants rated their impression of overall change in their migraine at 2 h after the initial dose compared with immediately before taking the study medication using the PGIC, a single-item, 7-point rating scale with responses ranging from 0 to 6 (0 = very much better, 1 = much better, 2 = a little better, 3 = no change, 4 = a little worse, 5 = much worse, 6 = very much worse). PGIC responders were those who reported a 0 (very much better) or 1 (much better). Responder definitions for the FDS, satisfaction with study medication, and the PGIC were based on qualitative interpretation of the respective response options.

### Statistical analyses

To improve precision in this smaller subgroup of TIRs, participant baseline data and responses to the PRO measures were pooled across the placebo arms of each trial and for the ubrogepant 50 mg treatment arms of ACHIEVE I and II. The ubrogepant 100 mg (ACHIEVE I) and 25 mg (ACHIEVE II) treatment arms were each included in a single trial only and not included in this pooled analysis [17, 18]. Efficacy analyses were based on the modified intent-to-treat (mITT) population. The mITT population included all randomized participants who received at least 1 dose of study medication, recorded a baseline migraine headache severity measurement, and had at least 1 measurement of migraine headache severity or a migraine-associated symptom at or before the 2-h postdose timepoint. The last observation carried forward approach was applied only to the FDS to impute missing post-baseline values. The analyses used a multivariable logistic regression model and all statistical tests were 2-sided tests performed at the 5% level of significance (*P* < 0.05). All analyses were conducted using SAS version 9.3 or 9.4 (SAS Institute Inc, Cary, North Carolina, USA). This was a post hoc analysis of prespecified endpoints, and we did not adjust for multiple comparisons; we report all statistical tests performed.

## Results

### Participants

**Fig. 1:**
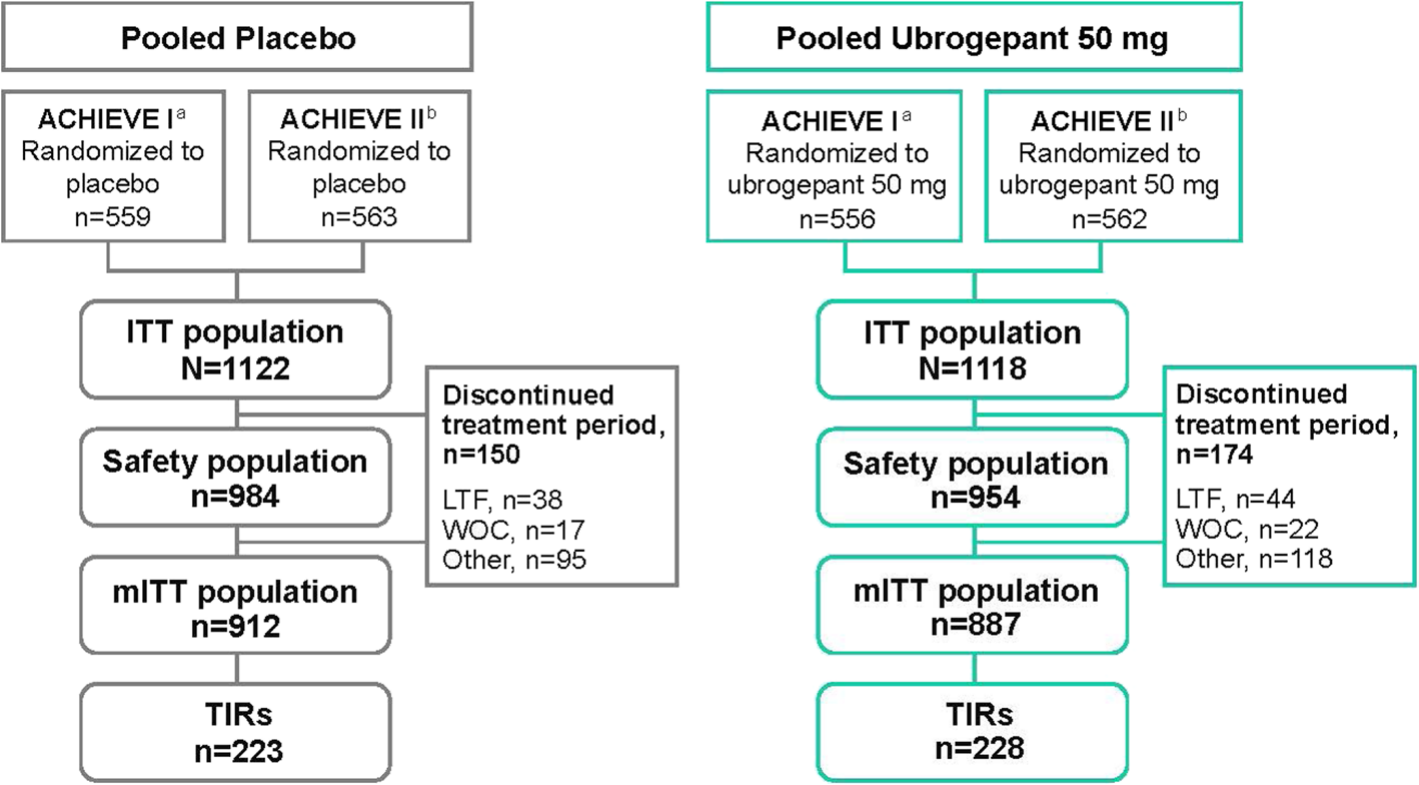
Participant flow diagram. LTF, lost to follow-up; ITT, intent-to-treat; mITT, modified intent-to-treat; TIR, triptan insufficient responder; WOC, withdrawal of consent. ^a^This analysis did not include the ubrogepant 100 mg arm from the ACHIEVE I trial. ^b^This analysis did not include the ubrogepant 25 mg arm from the ACHIEVE II trial

Overall, 1799 ACHIEVE participants were included in the pooled mITT population (*n* = 912 placebo, *n* = 887 ubrogepant 50 mg) (Fig. 1). Within this pooled population, there were 451 participants who met criteria for TIR. In total, 682 (38%) participants were categorized at baseline as triptan responders and 666 (37%) as triptan naive. Within the TIR subgroup, a majority (80%) of participants reported insufficient efficacy associated with triptan use, 17% reported insufficient tolerability, and 3% reported contraindications/warnings or precautions. Baseline demographics in the TIR subgroup were comparable between the pooled placebo and ubrogepant 50 mg treatment arms (Table 1).

**Table 1:** Participant characteristics of triptan insufficient responders (mITT Population)

	**Placebo**	**Ubrogepant 50 mg**
	**(*n* = 223)**	**(*n* = 228)**
Age, mean (SD), y	40.1 (10.7)	38.4 (10.7)
Female, *n﻿* (%)	206 (92.4)	214 (93.9)
White, *n* (%)	192 (86.1)	189 (82.9)
BMI, mean (SD), kg/m^2^	30.1 (7.9)	31.1 (8.4)
Moderate/high CV risk,^a^* n* (%)	32 (14.3)	24 (10.5)

*BMI* body mass index, *CV* cardiovascular, *mITT* modified intent-to-treat, *SD* standard deviation

^a^ ≥ 10% risk of CV disease within a 10-year period. CV disease risk assessed using an algorithm based on National Cholesterol Education Program (NCEP) risk factors and calculated Framingham Scores along with the presence of CV heart disease or other clinical forms of atherosclerotic disease, as well as diabetes

### Functional disability scale

**Fig. 2:**
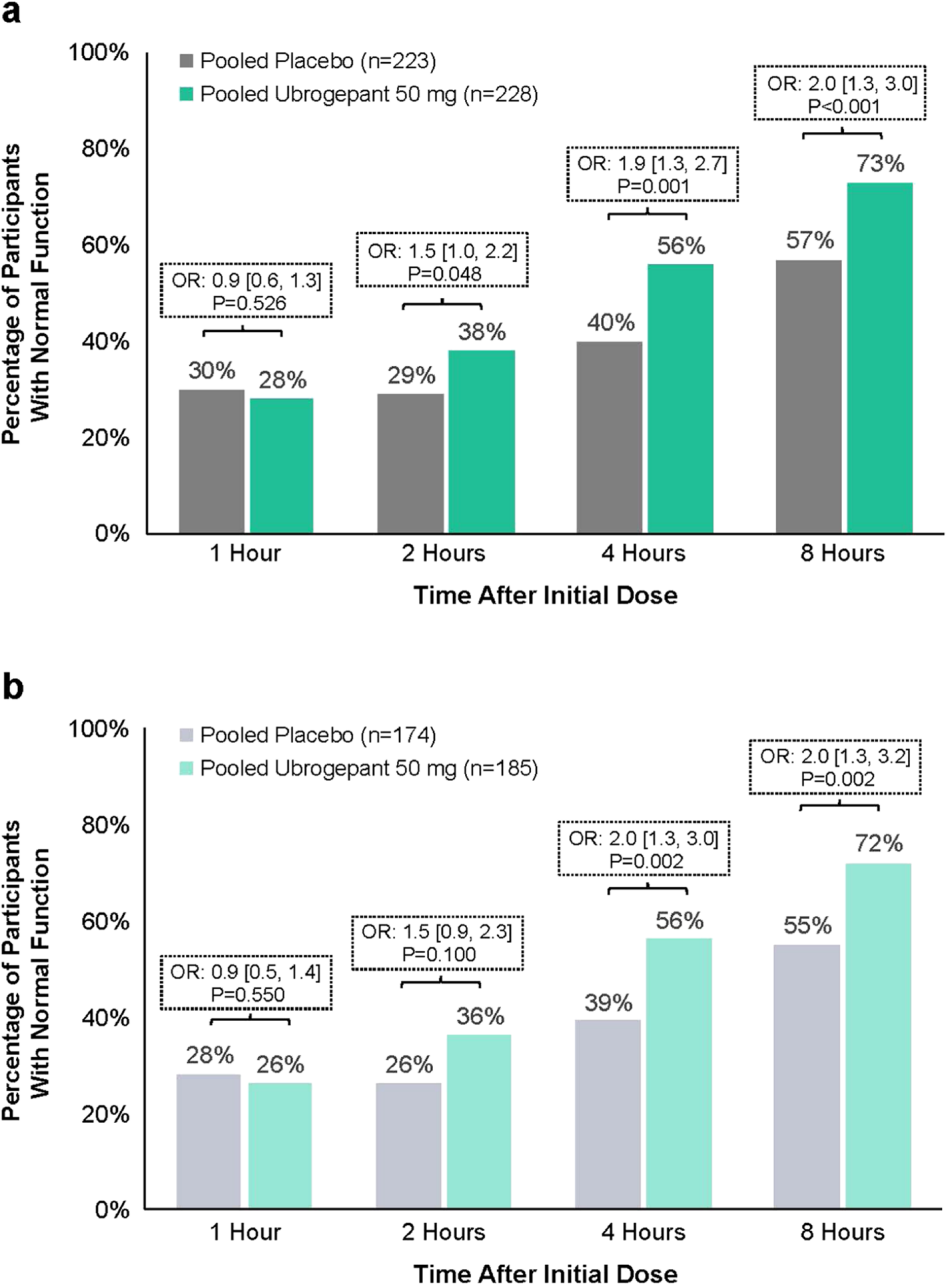
Proportion of participants reporting ability to function normally on the FDS in (**a**) TIR and (**b**) TIR due to insufficient efficacy groups. FDS, Functional Disability Scale; OR, odds ratio; TIR, triptan insufficient responder. *P*-values are based on logistic regression with treatment group, use of medication for migraine prevention, and baseline headache severity as covariates; last observation carried forward approach was used to input missing values post baseline

A significantly greater proportion of TIRs in the pooled ubrogepant 50 mg dose group compared with placebo reported that they had no disability (i.e., able to function normally) at 2 h (38% vs 29%; *P* = 0.048; odds ratio [OR]: 1.5 [95% confidence interval [CI]: 1.0, 2.2]), 4 h (56% vs 40%; *P* = 0.001; OR: 1.9 [95% CI: 1.3, 2.7]), and 8 h post dose (73% vs 57%; *P* < 0.001; OR: 2.0 [95% CI: 1.3, 3.0]) (Fig. 2a). Differences were not statistically significant at 1 h for ubrogepant 50 mg vs placebo (28% vs 30%; *P* = 0.526; OR: 0.9 [95% CI: 0.6, 1.3]). Results for disability were similar in the subgroup of TIRs who reported insufficient efficacy associated with triptans, with a significantly greater proportion of ubrogepant-treated participants compared with placebo able to function normally at 4 h (56% vs 39%; *P* = 0.002; OR: 2.0 [95% CI: 1.3, 3.0]) and 8 h (72% vs 55%; *P* = 0.002; OR: 2.0 [95% CI: 1.3, 3.2]) post dose (Fig. 2b). Results for this subanalysis were not significant at 1 or 2 h.

### Satisfaction with study medication

**Fig. 3:**
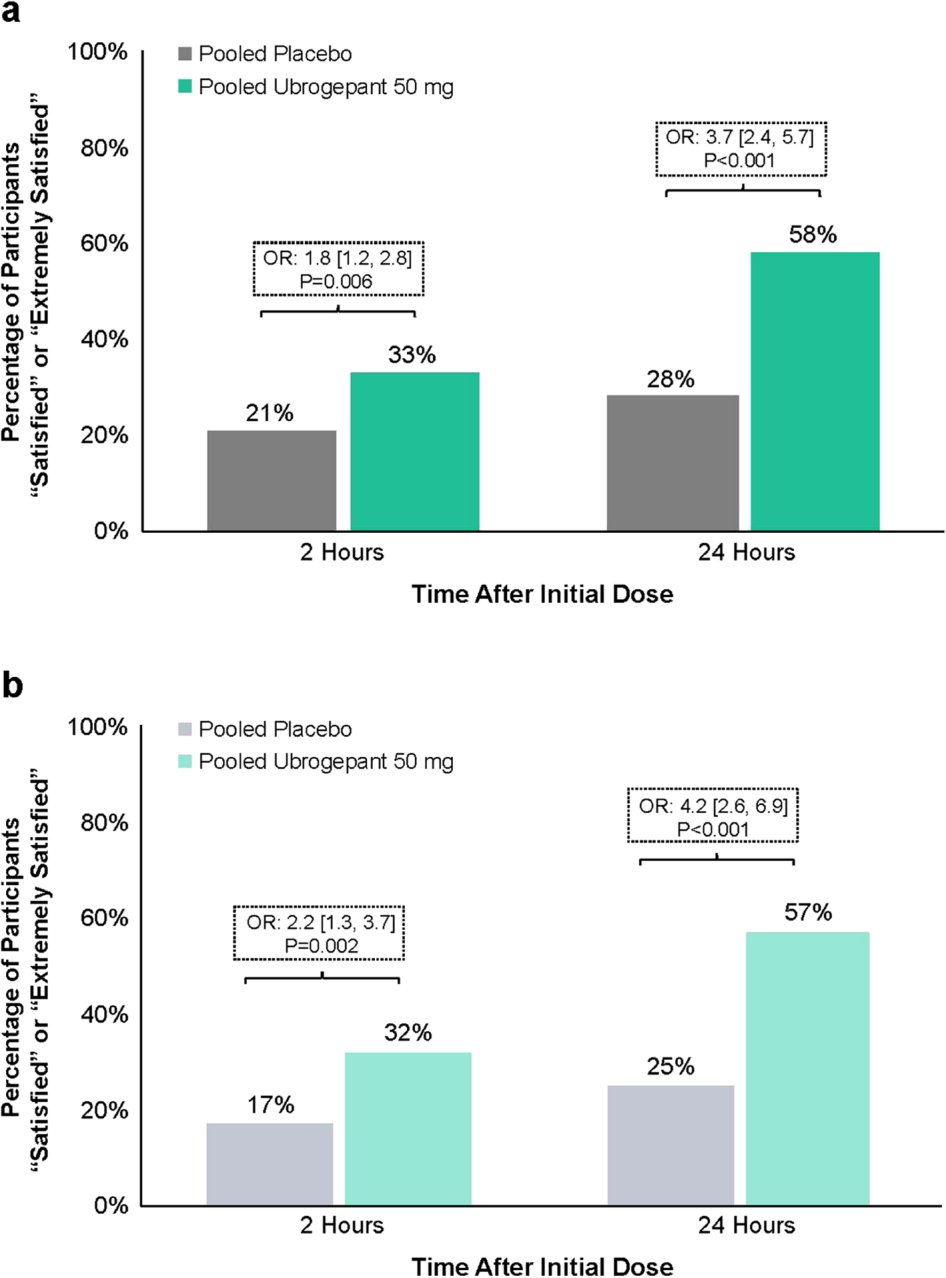
Proportion of participants “satisfied” or “extremely satisfied” with study medication at 2 and 24 h after initial dose in (**a**) TIR and (**b**) TIR due to insufficient efficacy groups. OR, odds ratio; TIR, triptan insufficient responder. *P*-values are based on logistic regression with treatment group, use of medication for migraine prevention, and baseline headache severity as covariates

Among TIRs treated with ubrogepant 50 mg, a significantly greater percentage (33%) reported being satisfied or extremely satisfied with study medication at 2 h post dose, compared with those treated with placebo (21%; *P* = 0.006; OR: 1.8 [95% CI: 1.2, 2.8]) (Fig. 3a). A significant difference was also observed at 24 h post dose, with 58% of ubrogepant 50 mg and 28% of placebo participants reporting being satisfied or extremely satisfied with study medication (*P* < 0.001; OR: 3.7 [95% CI: 2.4, 5.7]). Similar results were seen in the subgroup of TIRs who reported insufficient efficacy associated with triptans. In this TIR subgroup, a significantly greater proportion of ubrogepant 50 mg vs placebo participants reported being satisfied or extremely satisfied with study medication at both 2 h (32% vs 17%; *P* = 0.002; OR: 2.2 [95% CI: 1.3, 3.7]) and 24 h (57% vs 25%; *P* < 0.001; OR: 4.2 [95% CI: 2.6, 6.9]) post dose (Fig. 3b).

### Patient global impression of change

**Fig. 4:**
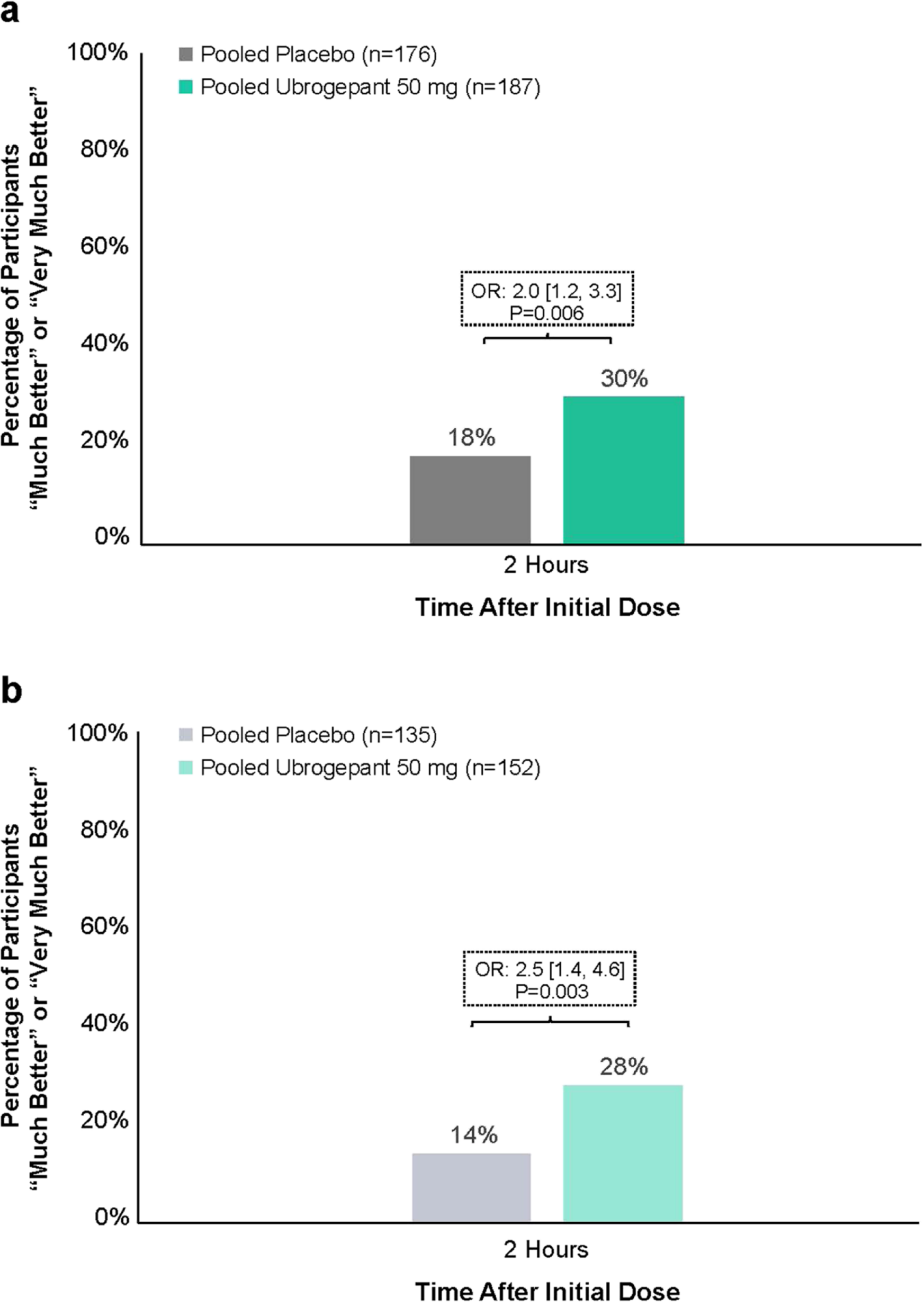
Proportion of participants reporting that their migraine was “much better” or “very much better” on the PGIC scale at 2 h after initial dose in TIR (**a**) and TIR due to insufficient efficacy (**b**) groups. OR, odds ratio; PGIC, Patient Global Impression of Change; TIR, triptan insufficient responder. *P*-values are based on logistic regression with treatment group, use of medication for migraine prevention, and baseline headache severity as covariates

In the group of TIRs, a significantly greater proportion of ubrogepant-treated participants compared with those in the placebo group reported the overall change in their migraine was “much better” or “very much better” on the PGIC at 2 h after the initial dose (30% vs 18%; *P* = 0.006; OR: 2.0 [95% CI: 1.2, 3.3]) (Fig. 4a). Within the TIR subgroup who reported insufficient efficacy associated with triptans, 28% of ubrogepant-treated and 14% of placebo participants reported the overall change in their migraine was “much better” or “very much better” on the PGIC at 2 h after the initial dose (*P* = 0.003; OR: 2.5 [95% CI: 1.4, 4.6]) (Fig. 4b).

### Safety

The incidence of treatment-emergent adverse events (TEAEs) and treatment-related TEAEs did not differ across historical triptan use subgroups. Among TIRs, 29 (12%) participants in the placebo group and 37 (15%) participants in the ubrogepant 50 mg group reported TEAEs within 48 h after the initial or optional second dose. No serious adverse events were reported in any group.

## Discussion

In this pooled, post hoc analysis, we compared ubrogepant 50 mg and placebo in those with an insufficient response to triptans. Many people with migraine will receive ubrogepant in clinical practice specifically because they are TIRs, thus characterizing outcomes in this subpopulation is clinically important [6, 26]. In the TIR group, ubrogepant-treated participants showed a significantly greater benefit over placebo with respect to function, satisfaction with study medication, and PGIC. The proportions of TIRs who reported that they had no disability and were able to function normally on the FDS were significantly greater in the ubrogepant 50 mg group than in the placebo group at 2, 4, and 8 h post initial dose. Results were generally similar in the subgroup of TIRs who reported insufficient efficacy associated with triptans, although statistical separation was present only at 4 and 8 h.

A significantly greater proportion of TIRs receiving ubrogepant vs placebo reported being satisfied or extremely satisfied with study medication at 2 and 24 h, and this was consistent in those who reported insufficient efficacy associated with triptans. In response to the PGIC, a significantly greater proportion of ubrogepant-treated TIRs and the insufficient efficacy subpopulation of TIRs reported that their migraine was “much better” or “very much better” at 2 h post initial dose compared with placebo. For this analysis, we conducted 8 statistical tests on the FDS and 5 were significant. We conducted 4 statistical tests on satisfaction and 2 on PGIC, all of which were significant. Of 14 total tests, 11 were significant and the endpoints that failed to reach significance were at early time points and in the smaller subgroup. We conclude that this pattern of results is unlikely to arise by chance. Patient-reported outcomes, such as ability to function, satisfaction with medication, and PGIC, can be clinically useful measures to assess treatment management in practice. While regulatory outcomes of pain freedom and absence of most-bothersome symptom provide standardized measures of efficacy in clinical trials, these outcomes can be challenging to apply in a real-world setting.

Low rates of persistence and adherence associated with triptans suggest a failure to meet acute treatment goals in many individuals with migraine [27, 28]. Individuals with migraine who are unable to attain an adequate acute treatment response with triptans represent a subgroup of people with substantial unmet need. Therefore, understanding clinically meaningful outcomes of treatment with ubrogepant in this population could help to inform clinicians’ and patients’ treatment decisions. The results of this analysis suggest that ubrogepant may be an effective acute treatment option and may help reduce migraine-related disability for individuals who are not adequately managed by triptans. No new safety signals were identified in this subgroup, further supporting the favorable tolerability profile of ubrogepant.

The coprimary efficacy outcomes in the ACHIEVE I and II trials were rates of pain freedom and absence of most bothersome symptoms at 2 h post dose, which were significantly greater in the ubrogepant 50 mg and 100 mg treatment arms compared with placebo [17, 18]. In a separate analysis of pooled data from the ACHIEVE trials, rates of pain freedom and absence of most bothersome symptom at 2 h with ubrogepant did not significantly differ in participants classified as triptan responders, TIRs, and triptan naive [29]. An additional analysis of ACHIEVE trial data showed a significant benefit of ubrogepant vs placebo in the overall trial populations on improvement in functional disability, satisfaction with study medication, and global impression of change [25]. The results of the current study expand these findings and further support the benefits of ubrogepant in people with migraine who are unable to achieve an adequate treatment response with triptans.

This analysis used pooled data from the placebo and ubrogepant 50 mg treatment arms of the ACHIEVE I and ACHIEVE II trials. Pooling the data from these trials allowed for a larger sample size within the subgroup of TIRs to improve overall precision and the ability to detect a difference in outcome due to treatment [30]. The ACHIEVE I and ACHIEVE II trials were identically designed, allowing for pooling across treatment arms that were shared across both studies. The ubrogepant 25 mg and 100 mg treatment arms were only included in a single study and were excluded from this post hoc analysis. This large sample size from the pooled ACHIEVE trials allowed for the subdivision of the population into sizable groups based on historical triptan experience; however, no formal sample size power estimation was done. Even with this larger pooled dataset, the sample size of TIRs who reported tolerability issues or contraindications was relatively small and statistical comparisons could not be made in these subgroups. Participants were categorized as TIRs based on their self-reported historical response to triptan use, and TIR status was not verified via a challenge study. The ACHIEVE trials themselves were randomized, double-blind, placebo-controlled studies with multiple outcome measures to assess the treatment effect of ubrogepant. Both trials evaluated the efficacy of ubrogepant on a single attack; therefore, no conclusions can be made concerning the impact of TIR status on repeated use of ubrogepant to treat multiple attacks. Additionally, no direct comparison regarding the efficacy between triptans and ubrogepant can be drawn from these data, as no active comparator was used in the ACHIEVE I and II trials.

## Conclusion

Collectively, data from the present study and the ACHIEVE I and II trials support the use of ubrogepant for the acute treatment of migraine, regardless of a person’s previous experience with triptans, and demonstrate the efficacy of ubrogepant in reducing the burden of a debilitating migraine attack. Beyond improved rates of pain freedom and pain relief from headache associated with a migraine attack, ubrogepant can restore the ability to function and is associated with patient satisfaction with treatment. In people with migraine who are TIRs, in comparison with the placebo arm, individuals treated with ubrogepant had favorable 2-h outcomes, as measured by the FDS, satisfaction with medication, and PGIC.

## Data Availability

AbbVie is committed to responsible data sharing regarding the clinical trials we sponsor. This includes access to anonymized, individual and trial-level data (analysis datasets), as well as other information (e.g., protocols and Clinical Study Reports), as long as the trials are not part of an ongoing or planned regulatory submission. This includes requests for clinical trial data for unlicensed products and indications. This clinical trial data can be requested by any qualified researchers who engage in rigorous, independent scientific research, and will be provided following review and approval of a research proposal and Statistical Analysis Plan (SAP) and execution of a Data Sharing Agreement (DSA). Data requests can be submitted at any time and the data will be accessible for 12 months, with possible extensions considered. For more information on the process, or to submit a request, visit the following link: https://www.abbvie.com/our-science/clinical-trials/clinical-trials-data-and-information-sharing/data-and-information-sharing-with-qualified-researchers.html.

## Abbreviations

CGRP: Calcitonin gene–related peptide
CI: Confidence interval
CV: Cardiovascular
FDS: Functional Disability Scale
HRQoL: Health-related quality of life
mITT: Modified intent-to-treat
OR: Odds ratio
PGIC: Patient Global Impression of Change
PRO: Patient-reported outcome
TEAE: Treatment-emergent adverse event
TIR: Triptan insufficient responder

## References

[1] Feigin VL, Vos T, Alahdab F, Amit AML, Bärnighausen TW, Beghi E et al (2021) Burden of neurological disorders across the US from 1990–2017: a global burden of disease study. JAMA Neurol 78(2):165–176. 10.1001/jamaneurol.2020.4152 PMC7607495 33136137

[2] GBD 2016 Disease and Injury Incidence and Prevalence Collaborators (2017). Global, regional, and national incidence, prevalence, and years lived with disability for 328 diseases and injuries for 195 countries, 1990–2016: a systematic analysis for the Global Burden of Disease Study 2016. Lancet.

[3] Headache Classification Committee of the International Headache Society (2018) The International Classification of Headache Disorders, 3rd edition. Cephalalgia 38:1–211 10.1177/0333102417738202 29368949

[4] Blumenfeld AM, Varon SF, Wilcox TK, Buse DC, Kawata AK, Manack A (2011). Disability, HRQoL and resource use among chronic and episodic migraineurs: results from the International Burden of Migraine Study (IBMS). Cephalalgia.

[5] Buse DC, Manack AN, Fanning KM, Serrano D, Reed ML, Turkel CC (2012). Chronic migraine prevalence, disability, and sociodemographic factors: results from the American Migraine Prevalence and Prevention study. Headache.

[6] American Headache Society (2019). The American Headache Society position statement on integrating new migraine treatments into clinical practice. Headache.

[7] Lipton RB, Munjal S, Buse DC, Alam A, Fanning KM, Reed ML (2019). Unmet acute treatment needs from the 2017 Migraine in America Symptoms and Treatment Study. Headache.

[8] Roberto G, Piccinni C, D’Alessandro R, Poluzzi E (2014) Triptans and serious adverse vascular events: data mining of the FDA Adverse Event Reporting System database. Cephalalgia 34:5–13 10.1177/0333102413499649 23921799

[9] Benemei S, Cortese F, Labastida-Ramírez A, Marchese F, Pellesi L, Romoli M (2017). Triptans and CGRP blockade - impact on the cranial vasculature. J Headache Pain.

[10] Gelfand AA, Goadsby PJ (2012) A neurologist’s guide to acute migraine therapy in the emergency room. Neurohospitalist 2:51–59 10.1177/1941874412439583 PMC3737484 23936605

[11] Imitrex [package insert] (2017) Research Triangle Park, NC: GlaxoSmithKline

[12] Dodick DW, Shewale AS, Lipton RB, Baum SJ, Marcus SC, Silberstein SD (2020). Migraine patients with cardiovascular disease and contraindications: an analysis of real-world claims data. J Prim Care Community Health.

[13] Lombard L, Farrar M, Ye W, Kim Y, Cotton S, Buchanan AS (2020). A global real-world assessment of the impact on health-related quality of life and work productivity of migraine in patients with insufficient versus good response to triptan medication. J Headache Pain.

[14] Marcus SC, Shewale AR, Silberstein SD, Lipton RB, Young WB, Viswanathan HN et al (2020) Comparison of healthcare resource utilization and costs among patients with migraine with potentially adequate and insufficient triptan response. Cephalalgia 40(7):639–649 10.1177/0333102420915167 PMC7273744 32223301

[15] Goadsby PJ, Holland PR, Martins-Oliveira M, Hoffmann J, Schankin C, Akerman S (2017) Pathophysiology of migraine: a disorder of sensory processing. Physiol Rev 97:553–622 10.1152/physrev.00034.2015 PMC5539409 28179394

[16] Ubrelvy [package insert] (2021) Madison, NJ: Allergan USA, Inc.

[17] Dodick DW, Lipton RB, Ailani J, Lu K, Finnegan M, Trugman JM (2019). Ubrogepant for the treatment of migraine. N Engl J Med.

[18] Lipton RB, Dodick DW, Ailani J, Lu K, Finnegan M, Szegedi A et al (2019) Effect of ubrogepant versus placebo on pain and the most bothersome associated symptom in the acute treatment of migraine: the ACHIEVE II randomized clinical trial. JAMA 322:1887–1898 10.1001/jama.2019.16711 PMC6865323 31742631

[19] Ailani J, Lipton RB, Hutchinson S, Knievel K, Lu K, Butler M (2020). Long-term safety evaluation of ubrogepant for the acute treatment of migraine: phase 3, randomized, 52-week extension trial. Headache.

[20] Curto M, Capi M, Cipolla F, Cisale GY, Martelletti P, Lionetto L (2020) Ubrogepant for the treatment of migraine. Expert Opin Pharmacother 21:755–759 10.1080/14656566.2020.1721462 32011192

[21] Do TP, Guo S, Ashina M (2019) Therapeutic novelties in migraine: new drugs, new hope? J Headache Pain 20:37 10.1186/s10194-019-0974-3 PMC6734360 30995909

[22] Singh A, Gupta D, Sahoo AK (2020) Acute migraine: Can the new drugs clinically outpace? SN Compr Clin Med 2:1132–1138

[23] Lucas C, Aly S, Touboul C, Sellami R, Guillaume X, Garcia G (2020) Patient-reported outcome in two chronic diseases: a comparison of quality of life and response profiles in severe migraine and severe asthma. Patient Relat Outcome Meas 11:27–37 10.2147/PROM.S222597 PMC7012635 32104124

[24] Houts CR, McGinley JS, Nishida TK, Buse DC, Wirth RJ, Dodick DW (2021). Systematic review of outcomes and endpoints in acute migraine clinical trials. Headache.

[25] Dodick DW, Lipton RB, Ailani J, Halker Singh RB, Shewale AR, Zhao S (2020). Ubrogepant, an acute treatment for migraine, improved patient-reported functional disability and satisfaction in 2 single-attack phase 3 randomized trials ACHIEVE I and II. Headache.

[26] Ailani J, Burch RC, Robbins MS (2021) The American Headache Society Consensus Statement: Update on integrating new migraine treatments into clinical practice. Headache 61:1021–1039 10.1111/head.14153 34160823

[27] Katic BJ, Rajagopalan S, Ho TW, Chen YT, Hu XH (2011) Triptan persistency among newly initiated users in a pharmacy claims database. Cephalalgia 31:488–500 10.1177/0333102410383058 20937605

[28] Messali AJ, Yang M, Gillard P, Tsai K, Tepper SJ, Bloudek LM (2014). Treatment persistence and switching in triptan users: a systematic literature review. Headache.

[29] Blumenfeld AM, Goadsby PJ, Dodick DW, Hutchinson S, Liu C, Finnegan M et al (2021) Efficacy of ubrogepant based on prior exposure and response to triptans: a post hoc analysis. Headache 61:422–429 10.1111/head.14089 PMC8252782 33749826

[30] Bangdiwala SI, Bhargava A, O’Connor DP, Robinson TN, Michie S, Murray DM et al (2016) Statistical methodologies to pool across multiple intervention studies. Transl Behav Med 6:228–235 10.1007/s13142-016-0386-8 PMC4927450 27356993

